# Is It Possible to Accurately Evaluate the Tumor Bed After Neoadjuvant Chemotherapy Using a 14G Tru-Cut Biopsy?

**DOI:** 10.3390/life15040604

**Published:** 2025-04-05

**Authors:** Berkay Kilic, Suleyman Bademler, Burak Ilhan, Inci Kizildag Yirgin, Ravza Yilmaz, Aysel Bayram, Hasan Karanlik

**Affiliations:** 1 Department of General Surgery, Oncology Institute, Istanbul University, Istanbul 34093, Türkiye; suleyman.bademler@istanbul.edu.tr (S.B.); karanlik@istanbul.edu.tr (H.K.); 2 Department of General Surgery, Istanbul Faculty of Medicine, Istanbul University, Istanbul 34093, Türkiye; burak.ilhan@istanbul.edu.tr; 3 Department of Breast Radiology, Oncology Institute, Istanbul University, Istanbul 34093, Türkiye; inci.kizildagyirgin@istanbul.edu.tr; 4 Department of Radiology, Istanbul Faculty of Medicine, Istanbul University, Istanbul 34093, Türkiye; ravza.yilmaz@istanbul.edu.tr; 5 Department of Pathology, Istanbul Faculty of Medicine, Istanbul University, Istanbul 34093, Türkiye; aysel.bayram@istanbul.edu.tr

**Keywords:** neoadjuvant systemic therapy, 14G tru-cut biopsy, false negative rate, radiologic complete response, pathologic complete response

## Abstract

**Background/Objectives:** Accurately identifying residual disease in the breast following neoadjuvant systemic therapy (NST) is a critical aspect of treatment planning. While surgery remains the standard treatment, its omission may be considered in exceptional responders. However, this strategy is still under investigation and carries local and distant recurrence risks. No definitive method currently exists to confirm pathologic complete response (pCR) after NST. This study evaluates the reliability of ultrasound-guided 14G Tru-Cut biopsy in assessing post-NST disease status. **Methods**: Data from 204 breast cancer patients who underwent ultrasound-guided 14G Tru-Cut biopsy before surgery at Istanbul University Oncology Institute (March 2015–May 2024) were analyzed. Concordance between Tru-Cut biopsy and final pathology was assessed, along with diagnostic accuracy parameters, including false-negative rate (FNR), accuracy, negative predictive value (NPV), and positive predictive value (PPV). **Results:** The median patient age was 45 years (range: 26–86). The median initial tumor size was 32 mm, reducing to 10 mm post-treatment. Pathologic complete response (pCR) was 33.8% in surgical specimens and 40.7% in biopsy samples. Biopsy misdiagnosed 15 patients, with an overall FNR of 11.1% and accuracy of 92.2% (95% CI, 7.1–18.1%; 95% CI, 87.6–95.5%). Among patients with radiologic complete response (rCR) (n = 99), FNR was 25.8%, and accuracy was 92.1%. The best outcomes were in the rCR and Tru-Cut pCR subgroup, with an FNR of 5.9% and accuracy of 95.6%. In triple-negative breast cancer patients, FNR was 5%, and an accuracy was 97.4%. **Conclusions:** Although obtaining eight or more samples with a 14G Tru-Cut biopsy after NST is insufficient to alter clinical practice for detecting residual disease, the promising results observed in the rCR and Tru-Cut pCR subgroups suggest its potential role in guiding treatment strategies.

## 1. Introduction

In recent years, there has been a growing trend in the utilization of neoadjuvant systemic therapy (NST) for the treatment of breast cancer [1,2]. This treatment approach provides systemic and regional control, offering patients the opportunity for personalized therapy. Additionally, reducing tumor size facilitates less invasive surgical intervention and helps eradicate micrometastatic disease, thereby decreasing the risk of spread [3]. However, the response to NST is heterogeneous, necessitating an objective assessment. The response is classified as either pathological complete response (pCR) or pathological partial response (pPR), depending on the presence or absence of residual tumor cells in the pathological examination of the surgical specimen [4].

Although NST has traditionally been used for patients with locally advanced breast cancer, it has become a routine practice due to increased rates of pathologic complete response (pCR), especially among those with triple-negative breast cancer (TNBC) and human epidermal growth factor receptor 2 (HER2)-positive breast cancer. Depending on the tumor stage, pCR rates of up to 67% have been observed in TNBC patients and up to 70% in HER2-positive breast cancer patients receiving combined chemotherapy and dual anti-HER2 therapy [5,6,7].

For women without residual disease after NST, surgery may not provide a therapeutic benefit, as invasive or in situ tumor cells may have been completely eradicated. However, breast surgery is still required to confirm whether any residual disease remains after NST.

Dynamic contrast-enhanced magnetic resonance imaging (DCE-MRI) is a valuable technique to evaluate residual disease following NST. A high correlation has been reported between MRI and pathology results in patients with locally advanced breast cancer, with a correlation coefficient of 0.75 [8]. However, the accuracy of MRI in detecting residual disease ranges from 63% to 93%, indicating that it is not sufficient as a standalone tool [9,10].

Recent studies have suggested that image-guided biopsy (IGBx) could be used as an alternative to surgery for diagnosing residual disease. Although initial reports [11,12] indicated a false negative rate (FNR) as high as 26-49%, recent studies have found the FNR to be as low as 3.2%, with an accuracy of 90% [13]. In a study, surgeons could detect residual tumors smaller than 0.5 cm when at least five samples were obtained, achieving an FNR of 0%, an accuracy of 90%, and a negative predictive value (NPV) of 100% [14].

In this study, we have aimed to evaluate the accuracy of 14-gauge (14-G) ultrasound-guided biopsy (UGBx) in predicting pCR or residual disease in breast cancer patients following NST to potentially omit surgery.

## 2. Materials and Methods

### 2.1. Patient Selection

The medical records of patients diagnosed with clinical T1-4N0-3M0 invasive primary breast cancer via image-guided core biopsy, treated with NST, and demonstrating either a radiologic partial response (rPR) or radiologic complete response (rCR) on imaging after NST were retrospectively reviewed from prospectively maintained electronic records at the Oncology Institute of Istanbul University between March 2015 and May 2020. Patients with any tumor size or histological subtype were eligible for participation. However, those with multicentric tumors, a history of ipsilateral breast cancer, marker dislocation, non-representative UGBx, or diffuse microcalcifications on imaging before NST were excluded. This study was approved by the medical ethics committee of Istanbul University (form number 20.02.2024-2434751), and written informed consent was obtained from all patients before surgery.

Standard diagnostic screening included breast ultrasonography (USG), mammography (MMG), and MRI at the time of diagnosis and after NST. The initial histological diagnosis of a malignant lesion was made using an ultrasound-guided (UG) 14G percutaneous core needle biopsy (CNB). Biopsies were performed using a free-hand technique with a 14G semi-automated core bi-opsy needle (Anjiotech, Medical Device Technologies, Florida, USA). Fine needle aspiration (FNA) was performed for any suspicious regional lymph nodes identified via sonography. Staining of 1% or more of tumor nuclei was defined as positive according to the recommendations of the American Society of Clinical Oncology and the College of American Pathologists [15]. The immunohistochemistry score +3 was defined as HER2-positive. The mammographic imaging protocol included craniocaudal, mediolateral oblique views, and additional views as requested by the interpreting radiologist. Dynamic contrast-enhanced magnetic resonance imaging examination was routinely used to evaluate treatment response before and after NST.

A location clip marker was placed in the center of the breast tumor under ultrasound guidance prior to the scheduled NST. For patients with multifocal tumors, clips were placed on the largest tumor. Neoadjuvant systemic treatment was administered based on institutional guidelines (anthracycline-based chemotherapy followed by a taxane). HER2-positive tumors were treated with trastuzumab and/or pertuzumab). Ultrasound-guided biopsies of the original tumor bed were performed after confirming the correct position of the marker using MMG or USG. The pathology results of the biopsies and surgical specimens were compared.

### 2.2. Response Evaluation

All patients underwent USG, MMG, and DCE-MRI after the last course of NST, with 71 patients using a 1.5-Tesla (T) scanner (Magnetom Aera, Siemens Healthineers, Erlangen, Germany) and 133 patients using a 3-T scanner (Achieva 3.0T, Philips Healthcare, Best, The Netherlands). Two breast radiologists (I.K, R.Y) reviewed all image findings with 5 and 12 years of experience, respectively. The clinical response was evaluated according to Response Evaluation Criteria in Solid Tumours (RECIST) 1.1 criteria. Radiological complete response was defined as the absence of any contrast-enhancing lesion on MRI and the complete disappearance of the tumor on mammography and breast USG. Radiologic partial response was defined as a 30% decrease in tumor size [16].

### 2.3. Biopsies After NST and Surgery

Ultrasound-guided biopsy and surgery were performed between the fourth and eighth weeks after the last course of chemotherapy. Since the surgery was performed in the same session, biopsies were obtained under general anesthesia in the operating room by breast radiologists from the periphery of the marker and/or tumor bed using a 14G needle under ultrasound guidance to minimize patient discomfort. The goal was to obtain at least eight biopsies from the 12 o’clock, 3 o’clock, 6 o’clock, 9 o’clock, and intermediate positions relative to the clip. The biopsy cavity and the clip placed before NST were localized with a double-hooked wire for subsequent surgery. The biopsy was categorized as non-representative if the histopathology failed to detect cancer or identify the tumor bed, and non-representative biopsies were excluded.

All patients underwent breast-conserving surgery or mastectomy, and sentinel lymph node and/or axillary dissection were performed following breast surgery. Histological response to chemotherapy was evaluated according to a modified version of the Scarff–Bloom–Richardson grading system. The absence of invasive or in situ focus in the pathological examination was defined as a pCR.

### 2.4. Statistical Analysis

A positive result for residual tumor was defined in both surgical specimens and UGBx. Conversely, the absence of viable tumor cells was a negative result. The primary endpoint was the determination of the FNR of image-guided biopsy after NST, while the secondary endpoints were accuracy, NPV, positive predictive value (PPV), specificity, and sensitivity. The FNR was defined as the proportion of cases where the imaging-guided biopsy did not detect a tumor (false negative). However, a tumor was subsequently identified in the surgical specimen (true positive). Continuous parameters were reported as median and range, while categorical parameters were expressed as percentages (%). The 95% confidence intervals, corresponding to the data following a binomial distribution, were computed using the standard approximation method. Statistical analysis was performed using Microsoft Excel and IBM Statistical Package version 21 (SPSS, Chicago, IL, USA).

## 3. Results

### 3.1. Patients and Tumor Characteristics in All Cohorts

This study included 204 patients with a median age of 45 (range 26–86). The median initial tumor size on pre-NST imaging (USG-MMG-MRI) was 32 mm (range 18–80 mm). Clinical T2 was the most common stage (73.1%), followed by cT3 (12.7%) and cT4 (8.8%) before NST. Two hundred-one (92.2%) patients were node-positive on axillary imaging. In total, 30 patients (14.7%) were HR+/HER2+, 38 (18.6%) were TNBC, 46 (22.6%) were pure HER2+, and 90 (44.1%) were HR+/HER2−. Invasive ductal carcinoma (IDC) (77.9%) was the most common cancer type, followed by invasive lobular carcinoma (ILC) (9.3%) and mixed-type carcinoma (3.4%). A total of 113 patients (55.4%) had grade 2 tumors, and 64 patients (31.4%) had grade 3 tumors. Post-NST imaging revealed a median tumor size of 10 mm (range 0–70 mm), with rates of rCR and rPR being 48.5% (n = 99) and 51.5% (n = 105), respectively (Table 1).

### 3.2. Pathology Results of the Surgical Specimen

A total of 69 patients (33.8%) were diagnosed with no residual tumor after neoadjuvant therapy (ypT0), indicating pCR, while 135 patients (66.2%) had residual disease in surgical specimens (6/135 with residual in situ, 129/135 with residual invasive disease). The pCR rate was highest in patients with pure HER2+ tumors (n = 27; 58.7%), followed by patients with HR+/HER2+ tumors (n = 17; 56.7%) and TNBC patients (n = 18; 47.4%).

The residual invasive disease was most commonly observed in HR+/HER2− patients (n = 82; 91.1%), with this subtype also exhibiting the most extensive residual invasive disease (70 mm). The lowest rate of residual invasive disease was in the pure HER2+ group (n = 15; 32.6%). The residual carcinoma in situ rate was highest in the pure HER2+ group (n = 4; 8.7%), while the triple-negative (TN) group had no residual in situ disease (n = 0). Residual in situ carcinoma was detected in one patient in the HR+/HER2+ and HR+/HER2− groups (Table 2).

### 3.3. Pathology Results of the Ultrasound-Guided Biopsy

After NST, UGBx was predominantly performed using a 14G needle from the tumor bed in the operating room just before surgery. The median number of cores obtained was 8 (range 4–12). Only four to six samples were collected in 27 patients (13.2%) due to technical problems, bleeding, or incompatibility. Eight or more representative biopsies were obtained in the remaining 177 patients (86.8%).

Ultrasound-guided biopsy correctly identified the pathologic status of the breast in 188 of 204 patients (true negative + true positive cases), achieving an overall accuracy of 92.2% (95% CI, 87.6–95.5%). In one patient (0.5%), a tumor was present in the biopsy specimen but absent in the surgical specimen pathology (false-positive case). Importantly, this case achieved both breast pCR and rCR and belonged to the HER2+ group at stage T2N1M0. Ultrasound-guided biopsy failed to identify residual disease in 15 patients (7.5%; false-negative cases).

No residual tumor was detected in 83 patients on UGBx. However, residual disease was found in the surgical pathology of 15 cases, resulting in an FNR of 11.1% (95% CI, 7.1–18.1%) and an NPV of 81.9% (95% CI, 73.8–88.1%). The lowest FNR among subgroups were 5%, 7.7%, 12.1, and 15.8% in TNBC, HR+/HER2+, HR+/HER2−, and HER2+ groups, respectively, (95% CI, 1.0–34.0%), (95% CI, 1.0–51.0%), (95% CI, 7.0–22.0%), and (95% CI, 6.0–46.0%).

The highest accuracy was achieved in the TNBC group (97.4%; 95% CI, 86.2–99.9%), followed by the HR+/HER2+ group (96.7%; 95% CI, 82.8–99.9%). Logistic regression analysis showed no significant association between UGBx and surgical histopathology regarding patient or tumor characteristics or biopsy technique (Table 2).

### 3.4. The False-Negative Cases 

Overall, 15 (7.4%) UGBx results were false-negative (FN). One case each was identified in the TNBC and HR+/Her2+ groups. The pre-NST tumor sizes were 42 mm and 35 mm, respectively, with a tumor stage of T2N1M0. Both achieved rCR, with residual invasive tumor sizes of 1.5 mm and 1 mm, respectively.

In the HER2+ group, three false-negative cases (6.5%) were observed, each with a residual tumor size of 1 mm. Initial tumor sizes in this group ranged from 25 to 53 mm, and all achieved rCR.

Ten false-negative cases were identified in the HR+/HER2− group. This group’s largest residual invasive tumor size was 12 mm, and six patients had a residual tumor diameter of less than 5 mm. The number of targeted biopsy samples collected was below the expected threshold in all FN cases (Table 3).

### 3.5. The rCR Group

A subgroup analysis was conducted to determine whether an optimal FNR value could be achieved. Patients without rCR were excluded, leaving 99 patients with rCR confirmed through a combination of USG, MMG, and MRI. Among these, 68 (68.7%) patients achieved pCR in surgical specimens. Residual disease was present in 31 (31.3%) patients, including 4 (4%) with residual ductal carcinoma in situ (DCIS) and 27 (27.3%) with residual invasive cancer.

Ultrasound-guided biopsy correctly diagnosed the breast pathology in 91 out of 99 patients (accuracy 92.1%, NPV = 89.3%, PPV = 100%) and failed to accurately identify the pathological status of the breast in 8 (8.1%) patients.

The FNR increased from 11.1% in the initial analysis to 25.8% (95% CI, 14.1–47.1%), while the accuracy rate remained similar, from 92.2% to 92.1% (95% CI, 84.7–96.5%) in the overall group. Subgroup FNRs also increased, ranging from 14.3% to 37.5%. Consequently, the targeted optimal FNR could not be achieved in the rCR group (Table 4).

### 3.6. The rCR and Tru-Cut pCR Group

A group of patients with no residual disease detected in UGBx and who achieved rCR was analyzed (n = 76). Eight false-negative cases were identified. The false-negative rate, accuracy, NPV, PPV, specificity, and sensitivity were calculated as 5.9%, 95.6%, 89.5%, 99.2%, 98.6%, and 94.1%, respectively, demonstrating improvements compared to other results. This subgroup analysis yielded the most favorable outcomes.

Among the false-negative cases, one patient each belonged to the TNBC and HR+/HER2+ groups, while three were from the HER2+ and HR+/HER2− groups. The residual tumor size was 5 mm in only one patient and smaller than 1.5 mm in the remaining seven patients. The number of biopsy samples collected ranged from four to seven. Overall, UGBx accurately detected residual disease measuring 5 mm or larger. Notably, UGBx performance was particularly effective in the TNBC and HER2+ groups, which are known to exhibit better responses to NST (Table 5).

## 4. Discussion

In this study, we evaluated the correlation between the ultrasound-guided 14G Tru-Cut biopsy from the tumor bed at the end of treatment and the final surgical pathology in 204 breast cancer patients who had localization clips placed before NST. Our goal was to identify patients who achieved pCR after NST to potentially avoid surgery. While our findings are promising, they do not fully support the accuracy of biopsy in detecting residual breast disease and/or pCR. In the initial analyses, we observed a low FNR of 11.1% and a high accuracy rate of 92.2% in the overall group. In the patient group where no residual disease was detected on biopsy and rCR was observed, biopsy performance was found to be improved, with an FNR of 5.9% and an accuracy of 95.6%. The best results were observed in the TN subgroup, where an FNR of 5%, an accuracy of 97.4%, an NPV of 94.7%, and a PPV and specificity of 100% were achieved. These findings demonstrate that although an ultrasound-guided Tru-Cut biopsy using a 14G needle to obtain eight or more samples from the clip-marked tumor bed after neoadjuvant chemotherapy may not definitively identify residual disease and/or pCR, it holds significant promise for selected patient groups in the future.

Understanding the factors influencing residual disease detection through biopsies is critical for designing future studies. This information is also essential for investigating the possibility of de-escalating breast surgery in patients who do not demonstrate residual malignancy after neoadjuvant chemotherapy. However, several international studies have reported alarmingly high false-negative rates in detecting pCR using minimally invasive techniques. The pCR rate varies significantly depending on tumor biology. A study evaluating 30 large meta-analyses has clearly demonstrated this variation. While the lowest response rates were observed in the hormone receptor-positive group, modifications in chemotherapy regimens led to higher pCR rates in the TN and HER2-positive groups. Moreover, the use of dual blockade in the HER2-positive group has resulted in the highest pCR rates [5,6,7,17].

The MICRA study [18] obtained representative biopsies from 156 of 167 patients using a 14G needle under ultrasound guidance. Although the rates of pCR and rCR were reported as 53.3% and 81.4% in the overall population, respectively, the FNR varied widely, ranging from 26% to 100%. Eight or more biopsy samples were obtained from 86% of the patients. The NOSTRA-PRELIM study [19] reported a false-negative rate of 22.2%, but this cohort consisted of a small group of patients (n = 20). In the four false-negative cases, the residual tumor size ranged from 0.5 to 9 mm, and only three biopsy samples were obtained from these patients. In the NRG-BR005 study, Basik et al. [20] reported the lowest false-negative rate in the HER2+ group (40%), while a relatively high rate was reported in the TN group (63.6%).

Our study achieved breast pCR in 69 (33.8%) patients in surgical pathology and 83 (40.7%) in Tru-Cut biopsy. The biopsy provided a correct diagnosis in 188 out of 204 (92.2%) patients, with 15 false negative cases. The characteristics of these cases were as follows: In the TN group, the biopsy failed to correctly identify only one patient. The initial tumor size was classified as T2N1, grade 2, with a Ki-67 of 80%. This patient had 1.5 mm of residual IDC, and six representative samples were taken. In the HR+/HER2+ group, the biopsy failed to identify one patient. The initial tumor stage was cT2N1, grade 2, Ki-67 = 40%, and 1 mm residual IDC was noted, with seven samples taken. Both groups achieved rCR. The FNR was the lowest in these two groups, at 5% and 7.7%, respectively. The accuracy rate was 97.4% in the TN group and 96.7% in the HR+/HER2+ group. On the other hand, no false positive cases were identified in these two groups, resulting in a PPV of 100%.

In the HR+ group, despite achieving rCR in six patients with residual disease measuring 5 mm or smaller, the biopsy failed to detect residual disease. However, the number of representative biopsy samples in these cases ranged from four to six. Conversely, in four out of 65 patients with tumors larger than 10 mm, the biopsy results were inaccurate. These patients did not achieve rCR, and fewer than six biopsy samples were obtained.

In the HER2+ group, the biopsy incorrectly identified residual disease in three patients. All these patients had a residual tumor size of 1 mm, and the number of biopsy samples was fewer than eight. Interestingly, in one case, the biopsy detected residual disease, but no residual tumor was found in the surgical pathology (false positive). We hypothesize that this discrepancy may have resulted from the minimal residual tumor sampled during the biopsy.

### Potential Reasons for Biopsy Failure

**Limitations of the 14G biopsy needle:** The 14G biopsy needle’s inability to retrieve the clip in the tumor bed may lead to missing small residual tumors near the clip. Our study aimed to obtain at least eight samples from the clip area, targeting the primary directions (12, 3, 6, and 9 o’clock) and the intercardinal directions (1, 4, 7, and 10 o’clock). Notably, none of the false-negative cases had eight or more samples collected. The potential of increasing the number of samples to reduce the FNR is promising. Excluding 27 patients, 177 out of 204 (86.8%) patients had eight or more samples taken, underscoring the significance of our research.

**Tissue amount:** Each sample obtained with the 14G Tru-Cut biopsy needle yields approximately 17.7 mg of tissue. In contrast, vacuum-assisted biopsy using 14G and 11G needles can yield approximately 36.8 mg and 94.4 mg of tissue, respectively [21,22]. Additionally, vacuum biopsy may allow for the retrieval of the clip, making it a viable alternative.

**Criteria for including rCR patients:** Including only patients who achieved rCR is not logical. In fact, within the group of patients who achieved rCR (n = 99), the FNR rates ranged from 14.3% to 37.5%. When the group with rCR was combined with those that did not show residual disease on Tru-Cut biopsy (n = 76), the overall FNR decreased significantly from 11.1% to 5.9%, demonstrating improved performance. Furthermore, after NST, DCE-MRI is superior to USG and MMG in assessing residual disease [13]. However, despite reported high specificity (85%) and sensitivity (80%), DCE-MRI alone is insufficient for a complete and accurate assessment [23,24]. Another challenge is the evaluation of persistent residual microcalcifications, as DCE-MRI is not entirely successful. Nevertheless, numerous studies have indicated no correlation between residual microcalcifications and residual disease, suggesting that these are often derived from necrotic material post-treatment or hematomas and fat necrosis following biopsies [25,26,27].

On the other hand, in line with recent advancements in imaging technology, we routinely incorporated a 3T scanner, in addition to USG and MMG, to assess treatment response before and after NST. Furthermore, the evaluation of treatment response and the performance of biopsies by breast radiologists with a minimum of five years of experience provided a significant advantage in ensuring accurate patient selection.

Although performing biopsies under general anesthesia may be considered a limitation, this approach helped alleviate patient anxiety, enabling radiologists to work more comfortably and increasing the rate of obtaining representative samples. We believe that these factors played a crucial role in reducing the FNR to an acceptable level of approximately 5%.

There are studies in the literature that demonstrate successful outcomes. Heil et al. [11] attempted to determine residual disease after NST in a cohort of 164 patients using core biopsy or vacuum-assisted biopsy (VAB) guided by ultrasound or mammography. Despite the reported high FNR, ranging from 42.1% to 64.7%, a notable finding was that among patients who underwent VAB guided by mammography (n = 16), the FNR was 0, and the NPV was 100%.

Kuerer et al. [28] reported a 5% FNR and 98% accuracy in 40 patients consisting solely of TN and HER2-positive cases by combining FNA and 9G VAB. After their study, the researchers concluded that obtaining 12 samples with 9G VAB was necessary to identify patients who demonstrated an extraordinary response after NST.

In the RESPONDER study [29], Heil et al. reported a 17.8% FNR with VAB performed exclusively under imaging guidance in early-stage (stage 1–2) breast cancer patients. When excluding patients with multicentric and suspicious microcalcifications on mammography, this rate dropped to 6.2%. Furthermore, when biopsies were performed using a 7G needle, an FNR of 0 was achieved. This study suggested that VAB could be accurately applied to assess pCR with appropriate patient selection and technical equipment. However, the practical feasibility of performing biopsies with a 7G needle remains a subject of debate. In another study involving 166 patients [30], after NST, in a group with 2 cm or smaller imaging abnormalities and taking six or more representative samples from the tumor bed, an FNR of 3.2% and an accuracy of 89.5% were reported. At the study’s conclusion, image-guided biopsy after neoadjuvant chemotherapy was predicted to define a pathological complex response in selected patients. In a meta-analysis examining nine studies evaluating the tumor bed after NST, it was stated that image-guided biopsies were not sufficiently successful in predicting pCR. The FNR ranged from 5-50%, the NPV ranged from 33.3–76.9%, and sensitivity rates varied between 50-82.2%. Moreover, no significant differences were observed between subgroups. Our study, however, was particularly notable in the triple-negative group, with an FNR of 5% [31].

A recent study demonstrated the use of artificial intelligence (AI)-guided VAB for detecting residual disease after NST. In this study, which involved 318 patients and employed AI algorithms, FNR ranged from 0% to 5.2%, and specificity ranged from 37.5% to 40.0% [32]. Additionally, an ongoing study at M.D. Anderson Cancer Center, which is expected to conclude in 2026, aims to provide insights into standardized procedures for detecting residual disease in patients who demonstrate an exceptional response to NST. In their early results, the authors reported that surgery was omitted in 31 patients (62%) who achieved a pCR through VAB and received radiotherapy, with no ipsilateral breast tumor recurrence observed during a median follow-up of 26.4 months [33]. Similarly, in the OPTIMIST trial from Korea, a prospective, multicenter, single-arm, non-inferiority study involving 533 participants, breast surgery was omitted after pCR was confirmed using MRI and VAB following NST. The 5-year disease-free survival rates for these patients are not expected to differ from those of patients who achieved pCR but underwent breast surgery. Patients with residual tumor size of 1 cm or less on MRI and a lesion-to-background signal enhancement ratio (L-to-B SER) of less than 1.6 were included. The results of these studies may challenge previously considered unchangeable rules, potentially leading to significant changes in breast surgery practice, just as similar findings have in the past [34].

Our study has several limitations, including its single-center, retrospective design and biopsies performed in the operating room under general anesthesia. Despite the limited patient cooperation, general anesthesia facilitated the acquisition of adequate surgical samples. Additionally, the number of patients in the subgroups was insufficient for making reliable prospective conclusions.

## 5. Conclusions

Combining imaging results obtained after NST with 14G Tru-Cut biopsies of eight or more samples from the tumor bed significantly improved residual disease detection, reducing FNR to as low as 5% and offering promising outcomes. However, reliably predicting pCR remains a challenging paradigm that has yet to be fully resolved. Studies utilizing vacuum-assisted core biopsy methods also present auspicious results.

Nevertheless, standardization, patient safety, and careful evaluation of long-term outcomes are crucial to successfully implementing these minimally invasive approaches. Future studies should focus on designing robust protocols for biopsy-based pCR prediction, considering these factors. Standardized procedures, advanced biopsy techniques, the incorporation of genomic profiling, and a multidisciplinary approach will further enhance the accuracy of biopsies in predicting pCR. Ultimately, these studies will significantly contribute to the de-escalation of surgical interventions.

## Figures and Tables

**Table 1:** Features of tumors by subtypes.

		Overall n = 204 (100%)	TNBC n = 38 (18.6%)	HER2+ n = 46 (22.6%)	HR+/HER2+ n = 30 (14.7%)	HR+/HER2− n = 90 (44.1%)
Age, (median)		45 (26–86)	45 (28–74)	46 (29–71)	44 (26–64)	45 (26–86)
Clinical tumor stage	T1	11 (5.4)	2 (5.3)	5 (10.9)	2 (6.7)	2 (2.2)
	T2	149 (73.1)	29 (76.3)	34 (73.9)	15 (50)	71 (78.9)
	T3	26 (12.7)	3 (7.9)	6 (13.1)	11 (36.6)	6 (6.7)
	T4	18 (8.8)	4 (10.5)	1 (2.1)	2 (6.7)	11 (12.2)
Clinical nodal status	N0	3 (7.8)	0	1 (2.2)	0	2 (2.2)
	N+	201 (92.2)	38 (100)	45(97.8)	30 (100)	88 (97.8)
NST	4 (AC + T)	117 (57.4)	25 (65.8)	22 (47.8)	16 (53.3)	54 (60)
	6 TAC	13 (6.4)	2 (5.3)	1 (2.2)	0	10 (11.1)
	Other	74 (36.2)	11 (28.9)	23 (50)	14 (46.7)	26 (28.9)
Initial tumor size on imaging, mm (median)		32 (18–80)	30 (18–45)	27 (20–46)	41 (20–65)	32 (18–80)
Tumor histology	IDC	159 (77.9)	30 (78.9)	35 (76.1)	26 (86.6)	68 (75.6)
	ILC	19 (9.3)	1 (2.6)	5 (10.8)	2 (6.7)	11 (12.2)
	Mixt	7 (3.4)	1 (2.6)	0	0	6 (6.7)
	Other	19 (9.4)	6 (15.9)	6 (13.1)	2 (6.7)	5 (5.6)
Ki-67	≤20	72 (35.3)	2 (5.3)	14 (30.4)	9 (30)	47 (52.2)
	>20–50	82 (40.2)	13 (15.9)	22 (26.8)	11 (13.4)	36 (43.9)
	>50	50 (24.5)	23 (46)	10(20)	10 (20)	7 (14)
Grade	1	27 (13.2)	0	4 (8.8)	1 (3.3)	22 (24.4)
	2	113 (55.4)	19 (50)	21 (45.6)	18 (60)	55 (61.2)
	3	64 (31.4)	19 (50)	21 (45.6)	11 (36.7)	13 (14.4)
LVI	None	96 (51.5)	18 (21.1)	20 (43.5)	14 (46.7)	44 (48.9)
	Present	108 (48.5)	20 (78.9)	26 (56.5)	16 (53.3)	46 (51.1)
MF on surgical specimen	Yes	29 (14.2)	3 (7.9)	2 (4.4)	4 (13.39	20 (22.2)
	No	175 (85.8)	35 8 (92.1)	44 (95.6)	26 (86.7)	70 (77.8)
rCR		99 (48.5)	25 (65.8)	34 (73.9)	22 (73.3)	18 (20)
rPR		105 (51.5)	13 (34.2)	12 (26.1)	8 (26.7)	72 (80)

TNBC = triple-negative breast cancer, HER2 = human epidermal growth factor receptor 2, HR = hormone receptor, NST = neoadjuvant systemic therapy, AC = adriamycin-cyclophosphamide, T = taxotere, TAC = taxotere, adriamycin, and cyclophosphamide, IDC = invasive ductal carcinoma, ILC = invasive lobular carcinoma, LVI = lymphovascular invasion, MF = multifocality, mm = millimeter, rCR = radiological complete response, rPR = Radiologic partial response.

**Table 2:** Pathology results of the surgical specimen and ultrasound-guided biopsy.

	Total n = 204 (%)	TNBC n = 38 (%)	HER2+ n = 46 (%)	HR+/HER2+ n = 30 (%)	HR+/HER2− n = 90 (%)
**In surgical specimen**					
No residual disease (ypT0 = pCR)	69 (33.8)	18 (47.4)	27 (58.7)	17 (56.7)	7 (7.8)
Residual disease	135 (66.2)	20 (52.6)	19 (41.3)	13 (43.3)	83 (92.2)
Residual in situ disease (ypTis)	6 (2.9)	0	4 (8.7)	1 (3.3)	1 (1.1)
Residual invasive disease	129 (63.3)	20 (52.6)	15 (32.6)	12 (40)	82 (91.1)
ypT1mic	15 (7.4)	3 (7.9)	5 (10.8)	2 (6.7)	5 (5.6)
ypT1a	17 (8.3)	4 (10.5)	3 (6.5)	1 (3.3)	9 (10)
ypT1b	17 (8.3)	2 (5.3)	3 (6.5)	3 (10)	9 (10)
ypT1c	31 (15.2)	2 (5.3)	2 (4.4)	2 (6.7)	25 (27.7)
ypT2+	49 (24.1)	9 (23.6)	2 (4.4)	4 (13.3)	34 (37.8)
**In UGBx**					
No residual disease, pCR	83 (40.7)	19 (50)	29 (63.1)	18 (60)	17 (18.9)
Residual disease	121 (59.3)	19 (50)	17 (36.9)	12 (40)	73 (81.1)
Residual in situ disease	11 (5.4)	1 (2.6)	5 (10.8)	3 (10)	2 (2.2)
Residual invasive disease	110 (53.9)	18 (47.4)	12 (26.1)	9 (30)	71 (78.9)
True negative cases, (n)	68	18	26	17	7
True positive cases, (n)	120	19	16	12	73
False negative cases, (n)	15	1	3	1	10
False positive cases, (n)	1	0	1	0	0
FNR, %	11.1	5	15.8	7.7	12.1
Sensitivity, %	88.9	95	84.2	92.3	87.9
PPV, %	99.2	100	94.1	100	100
NPV, %	81.9	94.7	89.7	94.4	41.2
Specificity, %	98.6	100	96.3	100	100
Accuracy, %	92.2	97.4	91.3	96.7	88.9

UGBx = ultrasound-guided biopsy, TNBC = triple-negative breast cancer, HER2 = human epidermal growth factor receptor 2, HR = hormone receptor, FNR = false negative rate, PPV = positive predictive value, NPV = negative predictive value, pCR = pathologic complete response.

**Table 3:** Details of false-negative cases.

Patient	Subtype	Clinical Stage and Tumor Size on MRI, mm	Grade	Histopathology in Surgical Specimen	Ki-67	Number of Cores, (n)	rCR
Patient 1	TNBC	T2N1, 42 *	2	1.5 mm, IDC	80	6	present
Patient 2	Her2+	T2N1, 25 *	3	1 mm, IDC	20	6	present
Patient 3	Her2+	T2N1, 34 *	3	1 mm, IDC	50	6	present
Patient 4	Her2+	T3N1, 53 *	2	1 mm, IDC	50	6	present
Patient 5	HR+/Her2+	T2N1, 35 *	2	1 mm, IDC	40	7	present
Patient 6	HR+/Her2−	T1N1, 18	1	5 mm, IDC	3	6	present
Patient 7	HR+/Her2−	T2N1, 22	2	1 mm, IDC	22	5	present
Patient 9	HR+/Her2−	T2N1, 25	2	10 mm, IDC	25	5	none
Patient 8	HR+/Her2−	T2N1, 22	2	5 mm, ILC	12	5	none
Patient 10	HR+/Her2−	T2N1, 25	2	10 mm, IDC	25	4	none
Patient 11	HR+/Her2−	T2N1, 28 *	2	1 mm, IDC	50	6	present
Patient 12	HR+/Her2−	T2N2, 22 *	2	1.2 mm, IDC	50	4	present
Patient 13	HR+/Her2−	T2N2, 25 *	2	5 mm, ILC	10	6	present
Patient 14	HR+/Her2−	T3N1, 55	2	10 mm, IDC	25	4	none
Patient 15	HR+/Her2−	T3N1, 55	2	12 mm, IDC	60	4	none

TNBC = triple-negative breast cancer, HER2 = human epidermal growth factor receptor 2, HR = hormone receptor; rCR = radiological complete response; mm = millimeter, MRI = magnetic resonance imaging, * = false negative cases in rCR + Tru-Cut pCR group.

**Table 4:** rCR group.

	Overall n = 99	TNBC n = 25	HER2+ n = 34	HR+/HER2+ n = 22	HR+/HER2− n = 18
pCR	68 (68.7)	18 (72)	26 (76.5)	17 (77.3)	7 (38.9)
Residual in situ disease	4 (4.1)	0	3 (8.8)	1 (4.5)	0
Residual invasive disease	27 (27.2)	7 (28)	5 (14.7)	4 (18.2)	11 (61.1)
True negative	68 (68.7)	18 (72)	26 (76.5)	17 (77.3)	7 (38.9)
True positive	23 (23.2)	6 (24)	5 (14.7)	4 (18.2)	8 (44.4)
False negative	8 (8.1)	1 (4)	3 (8.8)	1 (4.5)	3 (16.6)
False positive	0	0	0	0	0
FNR, %	25.8	14.3	37.5	20	27.2
Sensitivity, %	74.2	85.7	62.5	80	72.8
PPV, %	100	100	100	100	100
NPV, %	89.3	94.7	89.7	94.4	70
Specificity, %	100	100	100	100	100
Accuracy, %	92.1	96	91.2	95.5	83.3

pCR = pathologic complete response, FNR = false negative rate, PPV = positive predictive value, NPV = negative predictive value, TNBC = triple-negative breast cancer, HER2 = human epidermal growth factor receptor2, HR = hormone receptor.

**Table 5:** rCR and Tru-Cut pCR group.

	Primary Analysis		Subgroup Analysis	
Overall Group, n = 204	Residual Disease in Tru-Cut Bx (n = 121)	No Residual Disease in Tru-Cut Bx (n = 83)	Residual Disease in Tru-Cut Bx OR rPR (n = 128)	No Residual Disease in Tru-Cut Bx AND rCR (n = 76)
No residual disease in surgical specimen (n = 69)	1 (FP)	68 (TN)	1 (FP)	68 (TN)
Residual disease in surgical specimen (n = 135)	120 (TP)	15 **(FN)**	127 (TP)	8 **(FN)**
FNR, (%)	11.1		5.9	
NPV, (%)	81.9		89.5	
PPV, (%)	99.2		99.2	
Specificity, (%)	98.6		98.6	
Sensitivity, (%)	88.9		94.1	
Accuracy, (%)	92.2		95.6	

pCR = pathologic complete response, FNR = false negative rate, PPV = positive predictive value, NPV = negative predictive value, rCR = radiologic complete response, rPR = radiologic partial response, bx = biopsy. FN = false negative, TN = true negative, FP = false positive, TP = true positive.

## Data Availability

Due to patient confidentiality, the data presented in this study are available upon request from the corresponding author.

## Abbreviations

The following abbreviations are used in this manuscript

AI	Artificial intelligence
CNB	Core needle biopsy
DCE-MRI	Dynamic contrast-enhanced magnetic resonance imaging
DCIS	Ductal carcinoma in situ
FN	False negative
FNA	Fine needle aspiration
FNR	False negative rate
G	Gauge
HER2	Human epidermal growth factor receptor 2
IGBx	Image-guided biopsy
IDC	Invasive ductal carcinoma
ILC	Invasive lobular carcinoma
MMG	Mammography
NPV	Negative predictive value
NST	Neoadjuvant systemic therapy
pCR	Pathologic complete response
PPV	Positive predictive value
rCR	Radiologic complete response
RECIST	Response Evaluation Criteria in Solid Tumours
rPR	Radiologic partial response
T	Tesla
TN	Triple-negative
TNBC	Triple-negative breast cancer
UG	Ultrasound-guided
UGBx	Ultrasound-guided biopsy
USG	Ultrasonography
VAB	Vacuum-assisted biopsy
